# Antibacterial activity of human mesenchymal stem cells mediated directly by constitutively secreted factors and indirectly by activation of innate immune effector cells

**DOI:** 10.1002/sctm.19-0092

**Published:** 2019-11-08

**Authors:** Lyndah Chow, Valerie Johnson, Renata Impastato, Jonathan Coy, Alyssa Strumpf, Steven Dow

**Affiliations:** ^1^ Center for Immune and Regenerative Medicine, Department of Clinical Sciences Colorado State University Ft. Collins Colorado; ^2^ Gates Center for Regenerative Medicine, Department of Immunology and Microbiology University of Colorado Denver Aurora Colorado

**Keywords:** antibacterial, cytokines, infection, neutrophil, peptides, stem cells

## Abstract

Mesenchymal stem cells (MSC) have been shown to improve wound healing and suppress inflammatory immune responses. Newer research also indicates that MSC exhibit antimicrobial activity, although the mechanisms underlying this activity have not been fully elucidated. Therefore, we conducted in vitro and in vivo studies to examine the ability of resting and activated MSC to kill bacteria, including multidrug resistant strains. We investigated direct bacterial killing mechanisms and the interaction of MSC with host innate immune responses to infection. In addition, the activity of MSC against chronic bacterial infections was investigated in a mouse biofilm infection model. We found that MSC exhibited high levels of spontaneous direct bactericidal activity in vitro. Moreover, soluble factors secreted by MSC inhibited *Staphylococcus aureus* biofilm formation in vitro and disrupted the growth of established biofilms. Secreted factors from MSC also elicited synergistic killing of drug‐resistant bacteria when combined with several major classes of antibiotics. Other studies demonstrated interactions of activated MSC with host innate immune responses, including triggering of neutrophil extracellular trap formation and increased phagocytosis of bacteria. Finally, activated MSC administered systemically to mice with established *S. aureus* biofilm infections significantly reduced bacterial numbers at the wound site and improved wound healing when combined with antibiotic therapy. These results indicate that MSC generate multiple direct and indirect, immunologically mediated antimicrobial activities that combine to help eliminate chronic bacterial infections when the cells are administered therapeutically.

## Graphical abstract

Human bone marrow MSC secrete factors that interact synergistically with antibiotics to media direct bacterial killing. Activation of MSC enhances indirect mechanisms of bacterial killing in a mouse biofilm infection model, and facilitates increased anti bacterial activity of neutrophils.

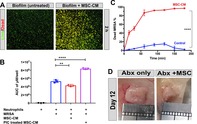

**Significance statement**

This study investigated the antimicrobial properties of human mesenchymal stem cells (MSC) and extends the results of previous studies by describing both the direct antimicrobial activity of MSC and the indirect antimicrobial effects mediated via interaction with host innate immune cells. This article describes in detail how MSC secreted factors augment the antimicrobial activity of nearly all classes of conventional antibiotics examined, including generating enhanced activity against drug‐resistant strains of *Staphylococcus aureus*. This study with human MSC serves to bridge previous studies and suggests the utility of systemic delivery of activated MSC for treatment of chronic drug‐resistant infection in human patients, in a scalable and clinically applicable manner.

## 1 INTRODUCTION

The increasing incidence of antibiotic resistant bacterial infections has prompted a search for more effective therapies, including alternatives to conventional antibiotics.^1^ Methicillin‐resistant *Staphylococcus aureus* (MRSA) in particular accounts for high mortality worldwide^2^ and is responsible for many chronic implant infections.^3^ The use of mesenchymal stem cell (MSC) to treat bacterial infections has received increasing attention in recent years, based on in vitro studies documenting direct bactericidal activity.^4^, ^5^, ^6^ Thus, it is possible that administration of MSC may be a means of potentiating conventional antibiotic therapy,^6^, ^7^ it is therefore important to elucidate more fully the mechanisms underlying MSC antimicrobial activity, both in vitro and in vivo.

Dealing with biofilm formation is currently one of the principle challenges facing clinicians when dealing with chronic infections. Bacteria in biofilms live in an environment that favors bacterial persistence and evasion of host immune responses.^8^ Within biofilms, bacteria elude killing from the immune system and antibiotics.^9^ For example, *S. aureus* biofilms can influence macrophage polarization and inhibit bacterial phagocytosis.^10^ The compact three‐dimensional structures of biofilms also limit neutrophil recruitment and killing because of a decrease in surface receptor recognition.^11^

Previous studies have shown that MSC have the ability to directly influence the immunological properties of macrophages and neutrophils by secreting factors such as PGE_2_,^12^ IL‐6, IL‐8, or IFN‐β.^13^ Following exposure to MSC‐secreted factors, macrophages develop increased phagocytosis, mediated in part by NADPH oxidase activation.^14^ Neutrophils exposed to MSC conditioned medium are resistant to apoptosis and demonstrate increased migration.^15^ Studies in animal models of infection have shown that human MSC can increase monocyte recruitment and decrease excessive neutrophil influx and neutrophil elastase production, particularly in murine models of cystic fibrosis and pulmonary *Pseudomonas aeruginosa* infection.^16^

MSC also produce antimicrobial peptides (AMPs), which are short peptides commonly found in neutrophils or epithelial cells.^17^ AMPs kill bacteria directly by disrupting the integrity of the microbial membrane,^17^ or by inducing the release of proinflammatory cytokines and in turn the recruitment of immune cells. Human MSC have been shown to produce multiple AMPs, including the cathelicidin peptide LL‐37^18^, hepcidin,^19^ β‐defensin 2, and lipocalin 2.^20^ MSC‐produced AMPs are thought to be one critical component regulating the ability of MSC administered therapeutically to control or eliminate bacterial infections, as explored in multiple animal models.^4^, ^18^, ^21^

A number of in vivo mouse models have explored the effect of MSC on acute bacterial infections. For example, human MSC decreased bacterial burden in a mouse model of *Escherichia coli* pneumonia,^18^ and also *Klebsiella pneumoniae* pneumosepsis.^22^ In another study, human MSC also reduced mortality associated with *P. aeruginosa* in a mouse peritonitis and sepsis model.^23^ MSC have also been shown to augment antibiotic treatment effects in murine cystic fibrosis,^24^ in part by the secretion of LL‐37.^16^ Lastly, instillation of MSC into airways of explanted lungs have been shown to decrease *E. coli* bioburden, and ameliorated acute lung injury including alveolar fluid clearance and inflammation.^25^

However, few studies to date have investigated whether MSC can also be used in the treatment of chronic bacterial infections. Notably, our group recently reported that activated murine MSC were effective for the treatment of chronic *S. aureus* biofilm infection in a mouse implant infection model.^4^ In this model, we found that activated MSC delivered systemically demonstrated strong antibacterial activity and elicited resolution of wound infection when combined with antibiotic therapy. Importantly, in the same study we also showed that in pet dogs with spontaneous multidrug resistant (MDR) wound infections, systemic administration of activated canine MSC cleared bacterial infection, even when administered to animals with infections that had persisted for months with antibiotic treatment alone.^4^

Based on these compelling data from realistic animal models of chronic bacterial biofilm infections, we have now investigated in greater detail the mechanisms by which human MSC may control or eradicate bacterial infections, for the ultimate goal of translating MSC therapy to humans with chronic infections. in vitro assays were used to investigate direct bacterial killing mechanisms, as well as indirect mechanisms involving host innate immune defense. In addition, a mouse chronic implant infection model was used to assess the effectiveness of activated human MSC. The results of these studies provide insights into the multiple mechanisms by which MSC may be used as adjunctive therapy along with antibiotics for treating highly drug‐resistant infections in relatively inaccessible sites such as implant infections. This information will help guide the design of clinical trials to investigate MSC therapy as a new tool for managing drug resistant infections.

## 2 MATERIALS AND METHODS

### 2.1 Generation of bone marrow‐derived stem cells

Human bone marrow aspirates (Lonza, Boston, Massachusetts) from healthy donors (three total used in this study) were plated in T75 tissue culture flasks (CellTreat Scientific Products, Pepperell, Massachusetts) with MSC media containing DMEM, 10%FBS (VWR, Radnor, Pennsylvania), Glutamax 1x, Pen/Strep 1x, NEAA 1x, Essential AA 1x (Thermo Fisher Scientific, Waltham, Massachusetts), and 2 ng/mL of human Recombinant Human FGF‐basic (PeproTech, Rocky Hill, New Jersey). Media changes were first performed on day 5, and the adherent cells were passaged starting day 12 using Trypsin‐EDTA (Thermo Fisher Scientific). BM‐MSC were then collected at low passage and stored in liquid nitrogen in freezing medium containing 9% DMSO and FBS for further use.

### 2.2 Bacterial culture

The MRSA strain of *S. aureus* (USA300) was kindly provided by H. Schweizer (Colorado State University). *E. coli* FDA strain Seattle 1946 (DSM 1103, NCIB 12210) was purchased from American Type Culture Collection (ATCC, Manassas, Virginia), Bacteria were propagated in LB broth (BD Falcon). Overnight cultures of bacteria were grown in MSC media without antibiotics prior to use in various assays. Sub cultures of bacteria were then grown to log phase also in MSC media (OD600 of 0.6, corresponding to 7.5 Log10 CFU/mL) prior to use.

### 2.3 Flow cytometric assessment of bacterial killing

Bacterial killing assessment using flow cytometry was performed according to manufacturer's instruction using LIVE/DEAD BacLight Bacterial Viability and Counting Kit (Thermo Fisher Scientific). Histograms were generated using FlowJo 10.5 software.

### 2.4 Direct bacterial killing assay (BKA)

BKA were performed as previously described.^4^ Conditioned medium (CM) from human BM‐MSC was generated by plating 5 × 10^5^ cells per well in a 24 well plate with 500 μL per well of antibiotic free media containing: DMEM, 10%FBS, Glutamax 1x, NEAA 1x, Essential AA 1x and FGF‐basic, then incubating at 37°C in a 5% CO_2_ incubator. Conditioned medium was collected 24 hours post plating and immediately frozen at −80°C. The CM was thawed prior to use and cellular debris was removed by centrifugation. For bactericidal activity assessment, 50 μL of log phase *S. aureus* cultures (OD600 of 0.6, corresponding to 7.5 Log10 CFU/mL) were inoculated in 500 μL of MSC CM in 24‐well plates. Cocultures of bacteria and CM were incubated at 37°C in ambient air for 3 hours, then numbers of viable bacteria were determined by plating log_10_ serial dilutions on LB agar 4 quadrant plates (Thermo Fisher Scientific) and manual counting of colonies 24 hours later according to previously published protocol.^26^ The ability of MSC CM to augment the bactericidal activity of major antibiotic classes was determined by incubating bacteria in MSC CM with the addition of subtherapeutic concentrations of antibiotics. The antibiotic concentrations to be used were determined in advance by titration and elucidation of minimal bactericidal concentrations.^26^ The antibiotic concentrations used in these assays were as follows: cefazolin (375 ng/mL), gentamicin (200 ng/mL), vancomycin (500 ng/mL), enrofloxacin (2 μg/mL), imipenem (30 ng/mL), and daptomycin (50 ng/mL). All antibiotics were obtained from Sigma‐Aldrich (St. Louis, Missouri). The interaction of MSC CM with antibiotics to induce bactericidal activity was evaluated for statistical significance using interaction factor calculations with two‐way ANOVA (Prism 5, GraphPad, San Diego, California). Synergy was defined as an increase in the bacterial killing percentage (MSC CM with antibiotics) that exceeded the MSC CM alone as well as the antibiotics alone, whereas additive effects were defined as a mean increase in bacterial killing of combined treatment vs single agents alone.

### 2.5 Detection of AMP expression by MSC using immunocytochemistry and flow cytometry

To determine intracellular expression of AMP by MSC, 10 000 cells were seeded on coverslips (Chemglass Life Sciences LLC, Vineland, New Jersey) in 24‐well cell culture plates overnight, then fixed with 4% PFA (Paraformaldehyde) (Fisher Scientific, Hampton, New Hampshire) for 10 minutes, washed with PBS and permeabilized with 0.1% Triton X‐100 (Sigma‐Aldrich,). Slides were then blocked using 5% v/v normal donkey serum and then incubated with primary antibodies, diluted appropriately. Primary AMP antibodies used include Anti‐Surfactant protein D antibody (ab203309 1:200 dilution), Anti‐Lipocalin‐2/NGAL antibody (ab63929), Anti‐beta 2 Defensin antibody (ab9871), Anti‐Hepcidin antibody (ab134790), Anti‐Cathelicidin antibody (ab180760) all at 1:100 dilution. All antibodies were purchased from Abcam (Cambridge, Massachusetts). Specificity controls for immunostaining included rabbit or goat IgG from nonimmune rabbits or donkeys, diluted to the same concentration as primary antibodies. Following primary antibody incubation, chambers were washed and incubated with secondary antibody donkey anti rabbit or anti goat Cy3 (Jackson ImmunoResearch Laboratories, Inc, West Grove, Pennsylvania) and counter stained with DAPI. Visualization of fluorescence staining was done using an Olympus IX83 spinning disk confocal microscope.

Flow cytometry to detect intracellular AMP expression was performed using the same antibodies, using saponin (0.15% in PBS) (Sigma‐Aldrich) with a 2‐hour permeabilization step after fixation. Samples were analyzed on a Beckman Coulter Gallios flow cytometer (Brea, California), and histograms were generated using FlowJo Software (Ashland, Oregon) v10.5.

### 2.6 MSC activation

MSC were incubated with Nod‐like receptor agonists and Toll‐like receptor agonists and a key pro‐inflammatory cytokine (interferon‐gamma) to elicit cell activation and to examine the impact on bactericidal activity and interaction with host innate immune responses. Cell activation was done using MSC suspended in 15 mL filter top Bio‐Reaction tubes (CellTreat Scientific Products) incubated in a CO_2_ incubator at 37°C. Prior to addition to tubes, MSC were detached from flasks by trypsinization and then resuspended at 2 × 10^6^ cells/mL in MSC growth medium without FGF.

Cells were treated with the following stimulants, at concentrations reported to be active in prior publications.^27^, ^28^, ^29^ *Stimulant 1*: γ‐D‐Glu‐mDAP (10 μg/mL) and a negative control muramyl dipeptide; *Stimulant 2*: type B CpG oligonucleotide 0.1 μM; *Stimulant 3*: poly‐inosinic, poly‐cytidylic acid (pIC) 10 μg/mL (InvivoGen, San Diego, California); *Stimulant 4*: lipopolysaccharide (10 ng/mL) (Sigma‐Aldrich). *Stimulant 5*: cytokine IFN‐γ (10 ng/mL) (PeproTech). For activation in vitro, MSC were incubated with stimuli for 2 hours at 37°C in a 5% CO_2_ incubator with agitation every 30 minutes, then washed with PBS and plated in 24‐well plates. Supernatants and cells were collected for assays 24 hours later.

### 2.7 Neutrophil bacterial phagocytosis assay

Neutrophils were collected from human blood using the Lympholyte‐Poly (Cedarlane, Peterborough, United Kingdom) separation gradient according to manufacturer's instructions. Studies were done using neutrophils obtained from three unrelated, healthy donors. Quantitative phagocytosis over time was performed using the IncuCyte ZOOM system (Essen BioScience Inc, Ann Arbor, Michigan). Log phase *S. aureus* cultures were first fixed and stained using the pHrodo Red Phagocytosis Particle Labeling Kit (Thermo Fisher Scientific) according to manufacturer's instructions. Neutrophils (5 × 10^5^) were incubated in 24‐well plate wells, with MSC growth media or MSC CM and *S. aureus* was added at an MOI of 25:1 (bacteria to cells). Images (9 per well) were collected every 15 minutes using a ×10 objective, and analyzed using IncuCyte S3 Software (Essen BioScience Inc).

### 2.8 Neutrophil extracellular trap assay

In all, 500 000 human neutrophils were plated on 0.01% poly‐L lysine (Sigma‐Aldrich) coated coverslips (Chemglass Life Sciences LLC) in 24‐well cell culture plates, then incubated with 0.5 mL MSC CM for 3 hours. Studies were done using neutrophils obtained from three unrelated, healthy donors. IRB approval was obtained for collection of human blood samples with signed informed consent. After washing off the CM, *S. aureus* was added at an MOI of 1 for the indicated time points in HBSS containing calcium, magnesium and 10% autologous human serum. Staining for neutrophil extracellular trap (NET) formation was performed according to a published protocol.^28^, ^30^ Cells were immunostained with antibodies for detection of histone H3 using Anti Histone H3 (RM188) Antibody (Caymen Chemical, Ann Arbor, Michigan) and neutrophil elastase Anti‐Neutrophil Elastase antibody (ab21595) (Abcam) with slight modifications for staining in a 24‐well plate. Images were taken on Olympus IX83 spinning disk confocal microscope, by imaging 15 random fields per condition. The NET area was calculated using ImageJ^31^; and the NET area (in pixels) was determined by merging channel 2 and 3 pixels (representing histone H3 and neutrophil elastase staining, respectively) then normalized to DAPI channel area.

### 2.9 RT‐qPCR analysis of AMP expression

To assess mRNA expression for AMPs, RNA isolated using the RNeasy kit (Qiagen, Hilden, Germany) per the manufacturer's instructions. The QuantiTect Reverse Transcription Kit (Qiagen) was then used to synthesize cDNA from 1 μg of RNA, again following the manufacture's protocol. Primers were designed using Primers Primer‐BLAST (NCBI) and concentrations of 100 and 200 nM were used for forward and reverse primers respectively. iQ SYBR Green Supermix (Bio Rad, Hercules, California) was used to detect fluorescence amplification on a Agilent Mx3000P QPCR System (Agilent Technologies, Santa Clara, California). Fold change was calculated using ddCT method^32^ normalized to untreated controls and housekeeping gene GAPDH. Primer sequences used are as follows:

Cathelicidin (LL37): Fwd 5′‐GAAGACCCAAAGGAATGGCC.

Rev 5′‐CAGAGCCCAGAAGCCTGAGC

 

Hepcidin: Fwd 5′‐CCCACAACAGACGGGACAAC.

Rev 5′‐ CTCCTTCGCCTCTGGAACAT

 

Lipocalin (LCN2): Fwd 5′‐GGAGCTGACTTCGGAACTAAAGG

Rev 5′‐TGTGGTTTTCAGGGAGGCC

 

Beta Defensin2 (hBD2): Fwd 5′‐CCAGCCATCAGCCATGAGGG

Rev 5′‐GGAGCCCTTTCTGAATCCGC

 

Surfactant Protein D (SPD): Fwd 5′‐ACAAAAAGAAACCTGCCATGCT

Rev 5′‐TGGGCATTGTTCTGTGGGAG

### 2.10 *S. aureus* biofilm assays

*S. aureus* (strain USA300) was grown to log phase in MSC growth media without antibiotics and FGF, then diluted to an optical density reading (O.D 600 nm) of 0.1. To generate biofilms, 200 μL of bacteria per well was incubated at 37°C in ambient air for 72 hours in triplicate wells of 96‐well flat bottom cell culture plates (Thermo Fisher Scientific). The impact of MSC CM on inhibition of biofilm formation was assessed by addition of 100 μL MSC CM to biofilm wells after 24 or 48 hours of biofilm formation. After 72 hours, nonadherent bacteria were removed by washing with PBS, and remaining biofilms were stained with 0.05% crystal violet solution (Sigma‐Aldrich). Retained crystal violet was then dissolved with ethanol and O.D readings were obtained from a microplate reader, using a wavelength of 570 nm.

To determine whether MSC CM could disrupt fully formed biofilms, 200 μL of MSC CM was added after aspiration of nonadherent bacteria to biofilm wells after the biofilms had been allowed to form for 72 hours. The biofilms were then incubated with MSC CM for lengths of time varying from 2 to 24 hours.

Live/dead visualization of biofilms was performed using the LIVE/DEAD BacLight Bacterial Viability and Counting Kit (Thermo Fisher Scientific) according to manufacturer's instructions, and visualized on using an Olympus IX83 confocal microscope. Ratios of live to dead cells were calculated by imaging total area of each channel using ImageJ software.

### 2.11 Detection of MSC secreted cytokines by ELISA

Supernatants were collected from BM MSC after 24 hours in culture, using same methods previously described for MSC CM collection, and IL‐8 and MCP‐1 concentrations were measured according to manufacturer's instructions using human DuoSet ELISA (R&D Systems, Minneapolis, Minnesota).

### 2.12 Mesh implant biofilm animal model

All procedures involving live animals were approved by the Institutional Animal Care and Use Committee at Colorado State University. *S. aureus* coated surgical mesh was implanted subcutaneously in *nu/nu* mice (Charles River Laboratories) as previously described.^4^ The mesh was coated with *S. aureus* (Xen36 strain) engineered to express luciferase (Caliper Life Sciences, Waltham, Massachusetts); and sites of bacterial infection were imaged using an IVIS in vivo imaging system (PerkinElmer Inc, Waltham, Massachusetts) every 3 days. Mice were treated by oral administration of amoxi‐clav (0.125 mg/mL, 0.03125 mg/mL in drinking water) combined with i.v. administration of pIC activated MSC (1 × 10^6^ cells in 200 μL DPBS per injection per mouse according to previously published dose^4^), given on 4 consecutive treatments at 3‐day intervals, starting at day 3 after implant insertion based on previous publication and preclinical trials.^4^ Sodium heparin (Fresenius Kabl USA, Lake Zurich, Illinois) was added at 100 IU/mL to MSC immediately prior to injection to prevent clumping. MSC from three separate donors were used for in vivo experiments (single donor for each independent experiment). MSC from all 3 donors were used at passage 4 (P4), with P1 defined as the first trypsinization at 12 days after plating whole bone marrow. All donor MSC were frozen at passage 2 at a concentration of 5 × 10^6^ cells/mL in freezing media containing 9% DMSO (Sigma‐Aldrich) in FBS. P2 cells were thawed at least 7 days prior to injection, expanded to passage 4 and injected immediately after trypsinization and washing with PBS. Population doubling (PD) was calculated as [time in culture * log (2)]/ [log(final cell count) − log(initial cell count)]. PD at P3 was calculated to be 23.6 hours SD ± 3.16 for n = 3 donor cell lines. PD at passage 4 for n = 3 donors 21.9 hours SD ± 10.3.

Photon flux for determination of bacterial density at the implant site was calculated using IVIS Living Image Software. For direct quantification of live bacteria present in the implant and surrounding tissues, tissues were excised at the completion of the study, weighed and then homogenized by sonication in PBS. Homogenates were then plated on LB agar quadrant plates (Sigma‐Aldrich) in log10 serial dilutions. *S. aureus* colonies were counted to determine CFU, 24 hours later.

### 2.13 Statistical analyses

Statistical comparisons between two treatment groups were done using nonparametric *t* tests (Mann‐Whitney test). Comparisons between three or more groups were done using one‐way ANOVA, followed by Tukey multiple means post‐test. Tests for synergy were performed using two‐way ANOVA with significant *P* ≤ .05 interaction factors denoting synergistic interactions.^33^ Synergy was defined as: an increased mean bacterial killing percentage of antibiotics with MSC‐CM compared with antibiotics alone or MSC alone. Analyses were done using Prism7 software (GraphPad, La Jolla, California). For all analyses, statistical significance was determined for *P ≤ .05, **P ≤ .01, ***P ≤ .001, ****P ≤ .0001.

## 3 RESULTS

### 3.1 MSC spontaneously produce factors with bactericidal activity

**Figure 1:**
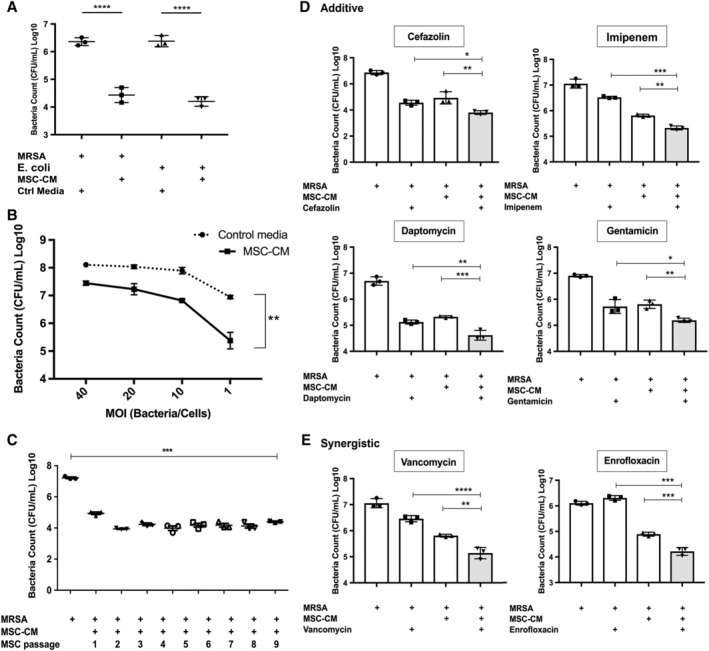
Direct antimicrobial activity of mesenchymal stem cells (MSC) and interaction with antibiotics in vitro. Conditioned medium (CM) from MSC was incubated with bacteria, as noted in the Materials and Methods section, to assess bactericidal activity. Data presented are representative of results obtained in three independent experiments using MSC from three different, unrelated donors. A, *S. aureus* and *E. coli* incubated with MSC CM. The y‐axis depicts bacterial colony counts (CFU/mL) in log scale. B, MSC CM incubated with increasing MOI of *S. aureus*. x‐axis shows depicts MOI, while dotted line represents bacterial growth when incubated with control media alone, solid line represents *S. aureus* CFU incubated with MSC CM. C, Bactericidal activity of MSC CM obtained from MSC at passages 1 through 9. For all figures statistical significance was determined for *P ≤ .05, **P ≤ .01, ***P ≤ .001, ****P ≤ .0001 as assessed by one‐way ANOVA and Tukey multiple means post‐test. Error bars depicting mean with SD in all panels. D, Interaction of MSC CM with antibiotics as expressed by bactericidal activity, using the bacterial killing assay (BKA) described in the Materials and Methods section. y‐axis shows bacterial count. Antibiotics with additive effect with MSC CM for bactericidal activity are depicted, including cefazolin, imipenem, daptomycin, and gentamycin. Gray Bars represent bacterial count with the addition of MSC CM and antibiotics. E, Two antibiotics (vancomycin and enrofloxacin) in which a positive synergistic interaction with MSC CM are depicted

The first studies were done to confirm that MSC produce antimicrobial factors spontaneously, as has been reported previously.^5^ We found that when *S. aureus* and *E. coli* were incubated with MSC CM, strong bactericidal activity was detected, and caused a decrease of greater than 2log CFU/mL of bacterial growth (Figure 1A). Moreover, incubation of a drug‐resistant strain of MRSA with MSC CM induced a 1.4‐fold decrease in bacterial CFU (Figure 1B). It should also be noted that the bactericidal activity of MSC CM was titratable (data not shown). This spontaneous bactericidal activity was observed using three different donor MSC (two male and one female; data not shown). Passage of MSC also did not alter bactericidal activity, at least for up to nine passages (Figure 1C). For example, MSC CM from passage nine cultures exhibited the same degree of bactericidal activity as passage 1 cultures, with up to 66% killing.

### 3.2 MSC CM acts synergistically with antibiotics to generate bactericidal activity

Previous studies have determined that the primary mediators of MSC secreted bactericidal activity are AMP.^5^ It has also been reported previously that AMP such as LL‐37 can synergize with conventional antibiotics for bacterial killing.^34^, ^35^ Therefore, we conducted studies to determine whether MSC CM contains factors that synergize with or exhibit additive bactericidal activity with conventional antibiotics, and to determine whether these effects are limited to only certain classes of common antibiotics.

First, we assessed bactericidal activity when subtherapeutic concentrations of six different classes of antibiotics were added to MSC CM, using *S. aureus* as target bacteria. We observed that four classes of antibiotics (cephalosporins, carbapenems, lipopeptides, and aminoglycosides) all exhibited additive bactericidal activity when combined with MSC CM (Figure 1D). Two other classes of antibiotics (glycopeptides and fluoroquinolones) exhibited synergistic activity with MSC CM (Figure 1E), with collective increase in bacterial killing up to 69%, which is greater than either MSC CM alone or antibiotics alone. Thus, these results indicated that MSC produced factors were capable of broadly enhancing the bactericidal activity of the most widely used classes of antibiotics.

### 3.3 Expression of AMPs by MSC

**Figure 2:**
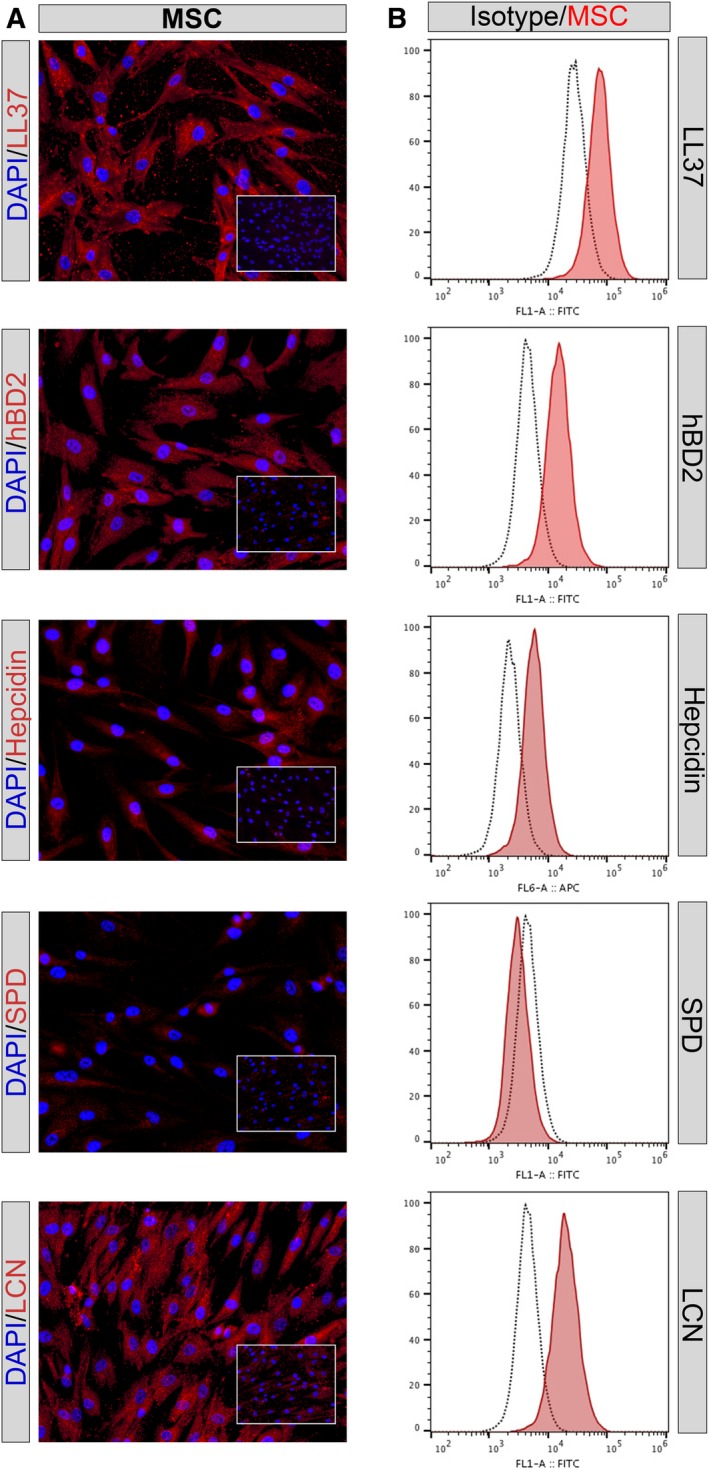
Intracellular expression of antimicrobial peptides assessed by immunocytochemistry (ICC) and flow cytometry. A, Mesenchymal stem cells (MSC) were immunostained with antimicrobial peptide antibodies, as described in the Materials and Methods section. Positive binding of antibodies to intracellular peptides is depicted as red staining. Immunostaining with matched irrelevant isotype antibodies are shown in bottom right inset boxes. Images shown in ×20 magnification. B, Intracellular immunostaining of antimicrobial peptides in MSC, cathelicidin LL‐37, beta defensin (hBD2), hepcidin, surfactant protein D (SPD), and lipocalin (LcN) as assessed by flow cytometry. Mean fluorescence intensity on x‐axis. Intracellular immunostaining with irrelevant control isotype matched antibody (black dotted line), positive staining in red. Figures are representative of results obtained using three different donor MSC

Expression of AMPs by human MSC has been reported previously.^5^, ^18^ To confirm the expression by the MSC used in our system, we examined expression of the following peptides cathelicidin LL‐37, beta defensin (hBD2), hepcidin, surfactant protein D (SPD), and lipocalin(LcN), using flow cytometry and immunocytochemistry.^5^ These studies revealed that MSC expressed four of the five AMPs, whereas the expression of surfactant protein D could not be conclusively detected by flow cytometry and only displayed low levels of staining by immunocytology (Figure 2A,B).

### 3.4 MSC activation by innate immune pathways alters AMP expression and cytokine production

**Figure 3:**
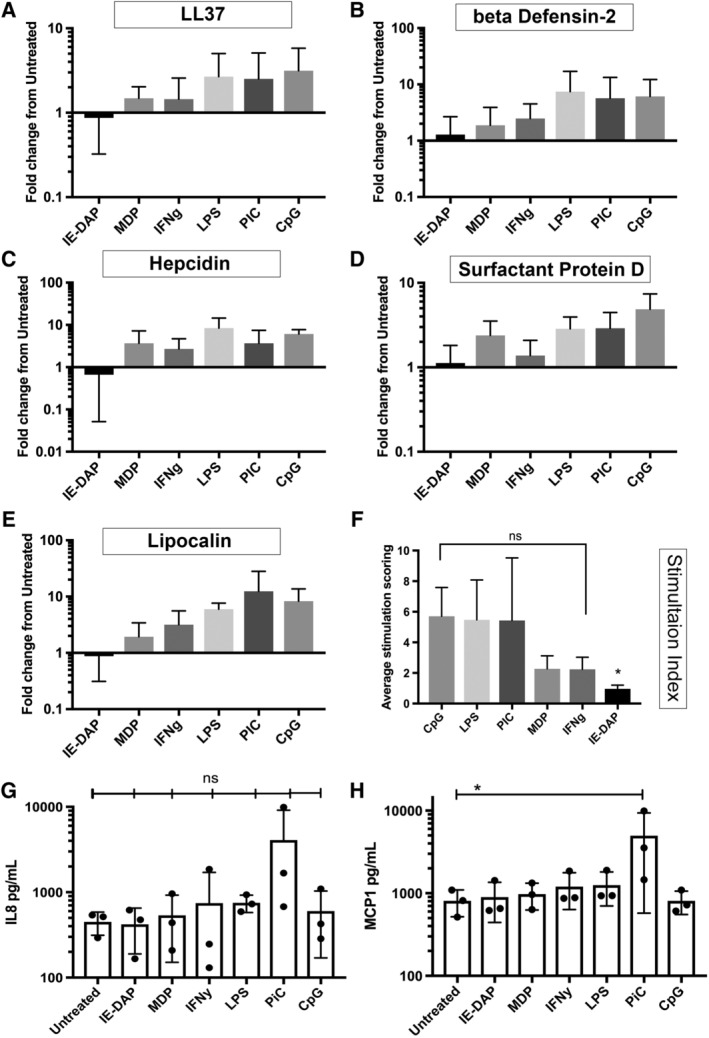
Effects of mesenchymal stem cells (MSC) activation with TLR ligands and cytokines on antimicrobial peptide (AMP) expression, as assessed by RT‐PCR. Gene expression in response to in vitro activation with stimuli, as noted in the Materials and Methods section. *Stimulant 1*: γ‐D‐Glu‐mDAP (IE‐DAP) and a negative control muramyl dipeptide (MDP); *Stimulant 2*: cytokine IFN‐γ (IFNg); *Stimulant 3*: lipopolysaccharide (LPS); *Stimulant 4*: poly‐inosinic, poly‐cytidylic acid (pIC). *Stimulant 5*: type B CpG oligonucleotide (CpG). The y‐axis depicts fold change in gene expression, calculated using ddCT method^32^ normalized to un‐stimulated MSC and housekeeping gene GAPDH. A, LL37 expression; B, beta defensin2 expression; C, hepcidin expression; D, surfactant protein D expression; E, Lipocalin. Figures depict average fold change in AMP expression, as assessed in three donor MSC. Error bars depicting mean of three technical replicates from three donor MSC with SD. F, Stimulation index depicting average fold changes of the expression of five AMP genes, ranked in order of most to least effective AMP upregulation stimuli. Error bars depicting mean with SD. G, IL‐8 secretion in response to activation. MSC were activated with the stimuli noted, then conditioned medium (CM) was collected 24 hours later and IL‐8 concentrations were determined using an IL‐8 ELISA, as noted in the Materials and Methods section. H, MCP‐1 secretion in response to MSC activation. MSC were activated with the stimuli noted, then CM was collected 24 hours later and MCP‐1 concentrations were determined using a specific ELISA, as noted in the Materials and Methods section. Each point on the cytokine graphs represents the mean of three technical replicates obtained from MSC generated from three different donors. * denotes *P* < .05 as assessed by ANOVA and Tukey multiple means post‐test

Previous studies have shown that activating MSC with innate immune stimuli increases their immune modulatory properties.^27^ In attempt to define their direct antimicrobial properties, we conducted studies to determine whether MSC activation through major innate immune pathways (Toll‐like receptors, NOD‐like receptors, cytokines) leads to an increase in factors associated with bactericidal activity (Figure 3A–E). We found that MSC stimulation by CpG oligonucleotides caused the greatest increase in expression of AMP genes (Figure 3F). However, none of the stimuli evaluated produced an actual increase in direct bactericidal activity (data not shown). We concluded therefore that MSC secretion of bactericidal factors (ie, AMPs) was primarily a constitutive process, and largely independent of cell activation status. Secretion of cytokines related to innate immune cell recruitment (ie, MCP‐1 and IL‐8) was however responsive to MSC activation, particularly by the TLR3 agonist pIC (Figure 3G,H). Thus, MSC activation appears to be more relevant to their interaction with host innate immune cells.

### 3.5 MSC factors trigger rapid bactericidal activity

**Figure 4:**
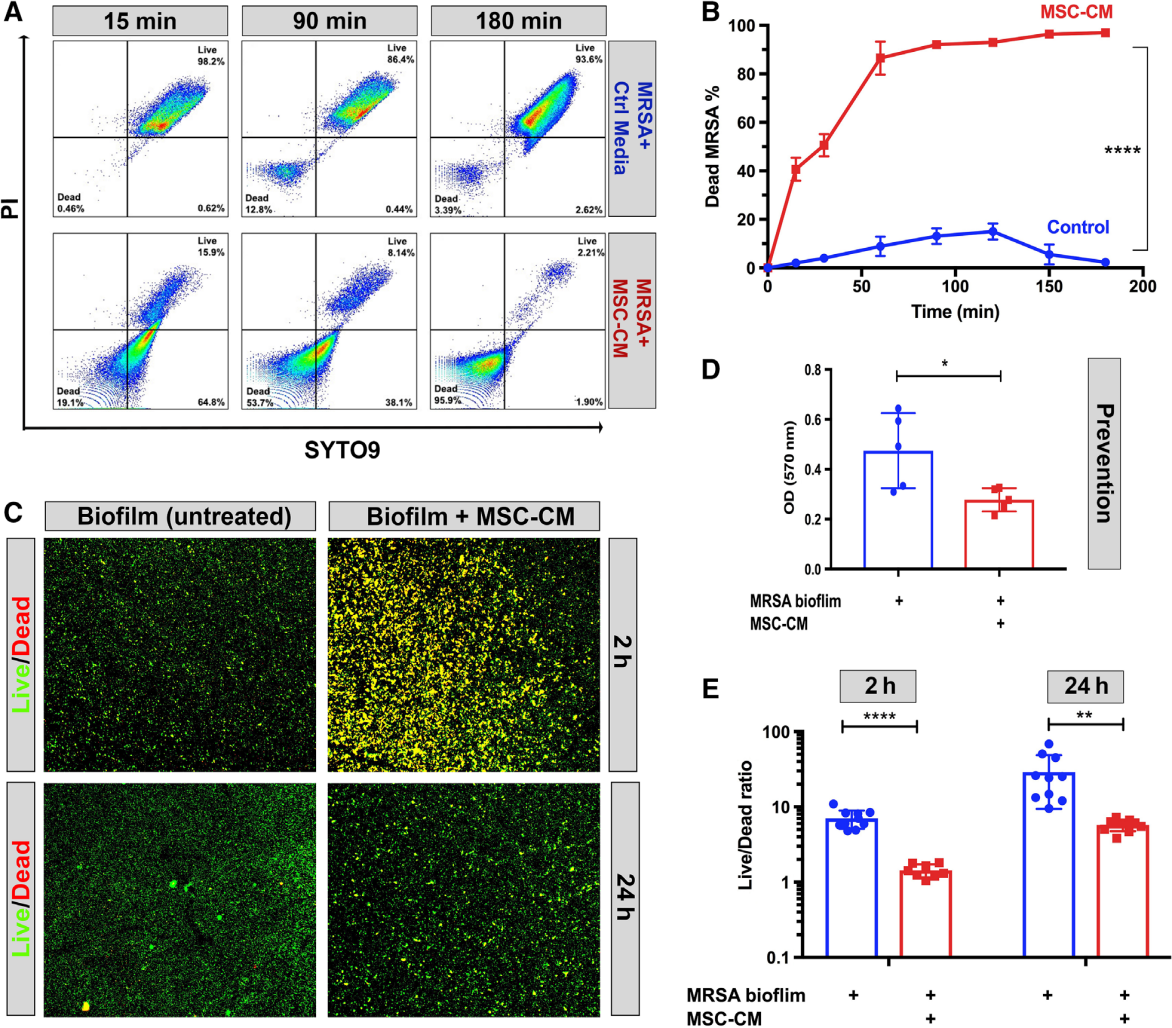
Killing of planktonic and biofilm bacteria by mesenchymal stem cells (MSC) conditioned medium (CM) as assessed by cell membrane permeability assay and immunocytochemical staining. A, Bacterial killing of *S. aureus* (USA‐300 strain) in the planktonic phase of growth was quantitated using the LIVE/DEAD BacLight kit for quantitation of bacterial cell membrane permeability, as noted in the Materials and Methods section. Representative flow cytometry plots from time points pretreatment, 15, 90, and 180 minutes after incubation with MSC CM. Dead and live quadrants are labeled in bottom left and top right. Figures are representations of results obtained from three different donor MSC CM. B, Percentage of dead bacteria as determined by flow cytometry at different time points, MSC CM incubated bacteria shown as the red line, whereas control medium depicted as blue line. Error bars represent mean and SD from three replicates. C, Bacteria (USA‐300) grown as biofilms were incubated with control medium and MSC CM for the indicated time points, then stained with LIVE/DEAD BacLight kit as described in the Materials and Methods section to identify live and dead bacterial colonies, as revealed by immunohistochemical staining and evaluation by confocal microscopy. Green (SYTO9) represents live bacterial clusters, whereas red clusters represent dead bacteria stained with propidium iodide. Merged channels show yellow color as red and green overlap. Right column “MSC‐CM” shows MRSA biofilm incubated for 2 (top) or 24 (bottom) hour with MSC conditioned medium. Left column “MRSA biofilm” grown in DMEM medium only with additives matched to MSC‐CM. Images taken with ×10 objective. 4D, Prevention of biofilm formation by MSC CM (compared with control medium) as assessed using *S. aureus* biofilm assays, as noted in the Materials and Methods section. The Y axis depicts bacterial colonies, quantitated using crystal violet staining after 72 hours in culture. Blue shows the biofilm grown in DMEM media with all additives, red shows biofilm with the addition of MSC CM. E, Effects of MSC CM on pre‐formed biofilms following 2 or 24 hours of exposure. Bars depict the ratio of live vs dead bacteria in biofilms, as quantitated using ImageJ software, as described in the Materials and Methods section. Statistical significance was determined for *P ≤ .05, **P ≤ .01, ***P ≤ .001, ****P ≤ .0001 as assessed by one‐way ANOVA and Tukey multiple means post‐test. Each experiment was conducted using CM from three different donor MSC. The figures depicted are representative of the results obtained in three independent experiments

We next asked the question of how rapidly MSC secreted factors could kill bacteria. Flow cytometry was used to assess bacterial cell membrane permeability (the first step in bacterial cell death). We observed that as soon as 15 minutes after exposure to MSC CM, bacterial cell death could be detected (Figure 4A). Bacterial death continued to increase for up to 3 hours, at which time 98% of the culture consisted of bacteria with disrupted membrane integrity (Figure 4B). Thus, MSC CM factors triggered bacterial killing extremely rapidly, consistent with what has been reported previously for AMP‐mediated killing.^4^, ^21^

### 3.6 MSC secreted factors disrupt biofilm formation

In addition to killing planktonic bacteria, factors secreted by MSC may also disrupt biofilm formation, as has been suggested previously.^4^ To examine this effect in greater detail, we determined whether MSC CM could prevent biofilm formation, or disrupt bacterial biofilms once they had already formed. We observed that addition of MSC CM prevented initial adhesion and formation of MRSA biofilms (Figure 4D). Importantly, addition of MSC CM also disrupted the biofilm when added to pre‐formed biofilms (Figure 4E) as evidenced by the decrease in the live/dead fluorescence ratio exhibited by bacteria following exposure to MSC CM. The effects of MSC CM persisted for up to 24 hours from the time of addition, with a 79% significant decrease in live/dead florescent ratio observed in biofilm cultures 24 hours after addition of MSC CM (Figure 4E). The effect of MSC CM on disruption of bacterial viability in established biofilms could also be readily visualized by confocal imaging (Figure 4C). Thus, MSC secrete factors that disrupt both biofilm formation and kill bacteria in already established biofilms, which may account in part for the effectiveness of MSC at controlling and eliminating biofilm infections associated with implants, as we previously reported.^4^

### 3.7 Activated MSC CM stimulates neutrophil phagocytosis

**Figure 5:**
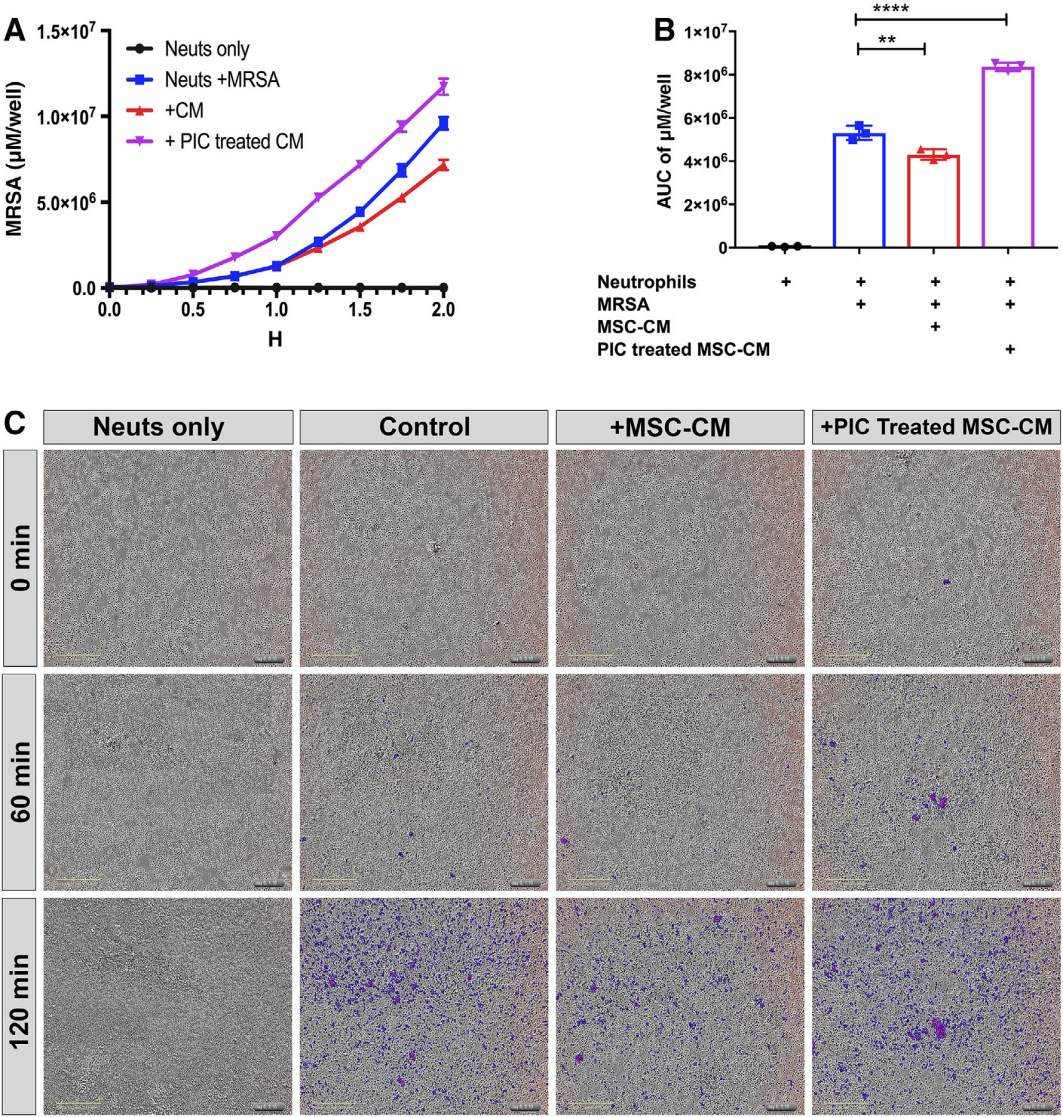
Effects of mesenchymal stem cells (MSC) conditioned medium (CM) on neutrophil phagocytosis. A, Neutrophils obtained from healthy donors were incubated with MSC CM to assess the effects on bacterial phagocytosis, using methicillin‐resistant *S. aureus* (MRSA) bacteria labeled with the pH red dye, as described in the Materials and Methods section. The x‐axis depicts time of incubation of neutrophils with bacteria (hours), while the Y axis depicts phagocytosed bacteria, quantitated as the average μM per well. Color legend for each conditions are shown in top right. B, Area under curve calculations of total phagocytosed bacteria over a 2‐hour time period are depicted. Control neutrophils (no bacteria) depicted in black, control neutrophils with bacteria only (blue), neutrophils incubated with MSC CM (red), and neutrophils incubated with pIC (Poly I:C) activated MSC CM (purple). The AUC was calculated using the Prism 7 software. Statistical significance was determined for *P ≤ .05, **P ≤ .01, ***P ≤ .001, ****P ≤ .0001 as assessed by one‐way ANOVA and Tukey multiple means post‐test. C, Representative photos obtained from the IncuCyte ZOOM system from neutrophils incubated for 0, 1, and 2 hours following addition of labeled *S. aureus*, for each of the four conditions tested. Purple indicates phagocytosed bacteria. Scale bar shown in yellow in bottom left corner of each panel

We next investigated the response of neutrophils to exposure to MSC CM with respect to altering phagocytosis, as described in the Materials and Methods section. Neutrophils were incubated with CM from resting MSC or with CM from pIC activated MSC, and bacterial phagocytosis was quantitated (Figure 5A,B). Neutrophils incubated with CM from pIC‐activated MSC exhibited significantly increased bacterial phagocytosis, compared with control neutrophils or neutrophils incubated with nonactivated MSC CM (Figure 5C). It is apparent then that activation of MSC by a TLR ligand induces increased secretion of a factor or factors that stimulate greater neutrophil phagocytosis of bacteria, an effect that could help in eradication of chronic infections in vivo.

### 3.8 Treatment with activated MSC CM increases NET area

**Figure 6:**
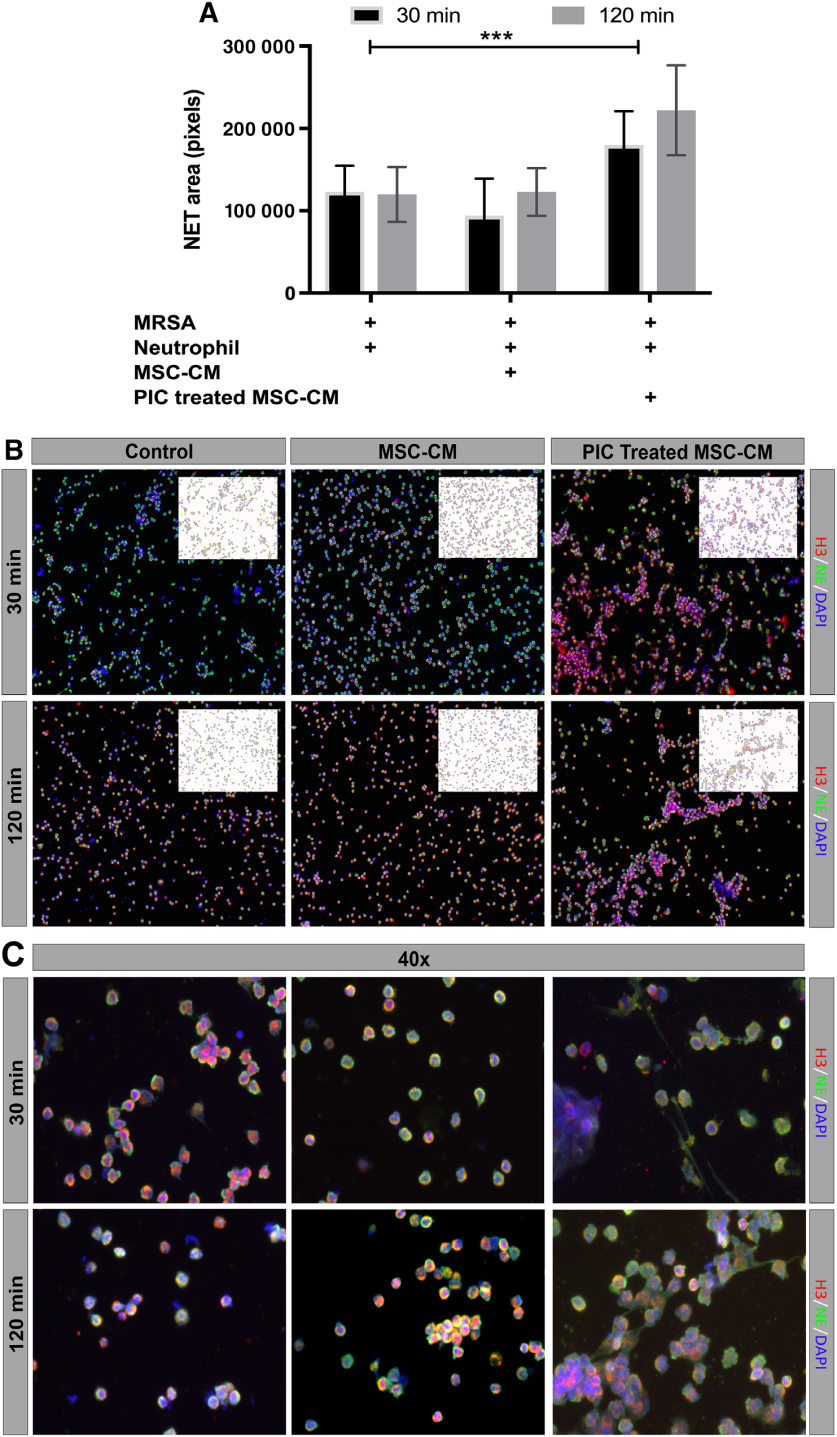
Effects of mesenchymal stem cells (MSC) conditioned medium (CM) on neutrophil extracellular trap (NET) formation. A, Neutrophils were incubated with MSC CM or medium, then incubated with live *S. aureus* for 30 minutes or 2 hours, as noted in the Materials and Methods section, then fixed and immunostained for detection and quantitation of NET formation, using confocal microscopy. Total NET area was normalized to DAPI cell count, and was digitized and quantitated using ImageJ software, as described in the Materials and Methods section. Bars depict the total area at 30 minutes (black) or 2 hours (gray) following exposure to *S. aureus*. *** denotes *P* < .0005 as assessed by ANOVA and Tukey multiple means post‐test. Each experiment was conducted using MSC CM obtained from three different donor MSC, and neutrophils were collected from three unrelated healthy donors. B, Representative ×10 magnification images of NET formation by neutrophils 30 minutes (top row) or 2 hours (bottom row) after exposure to *S. aureus*. Red, green, and blue depict histone H3, neutrophil elastase, and DAPI expression, respectively. The upper right corner of each image depicts shows NET total area, calculated by Image J software, with colors inverted for clarity. C, Representative ×40 magnification images of neutrophil NETs, imaged under same conditions as described for (B)

NETs are produced by neutrophils following contact with bacterial products and certain pro‐inflammatory cytokines; and are an important mechanism by which neutrophils can kill bacteria and prevent their spread into tissues. We observed that when neutrophils were incubated with CM from activated MSC, there was a significant increase in the total NET area produced per cell after contact with *S. aureus* (Figure 6A). The activated MSC also induced significantly greater NET area formation than CM from nonactivated MSC. The effects of MSC CM on NET formation were detected as soon as 30 minutes after exposure (Figure 6B,C). Thus, induced NET formation is another mechanism by which MSC could stimulate the innate immune system to increase bacterial elimination and chronic infection control.

### 3.9 Treatment with activated MSC decreases bacterial burden in a mouse model of chronic biofilm infection

**Figure 7:**
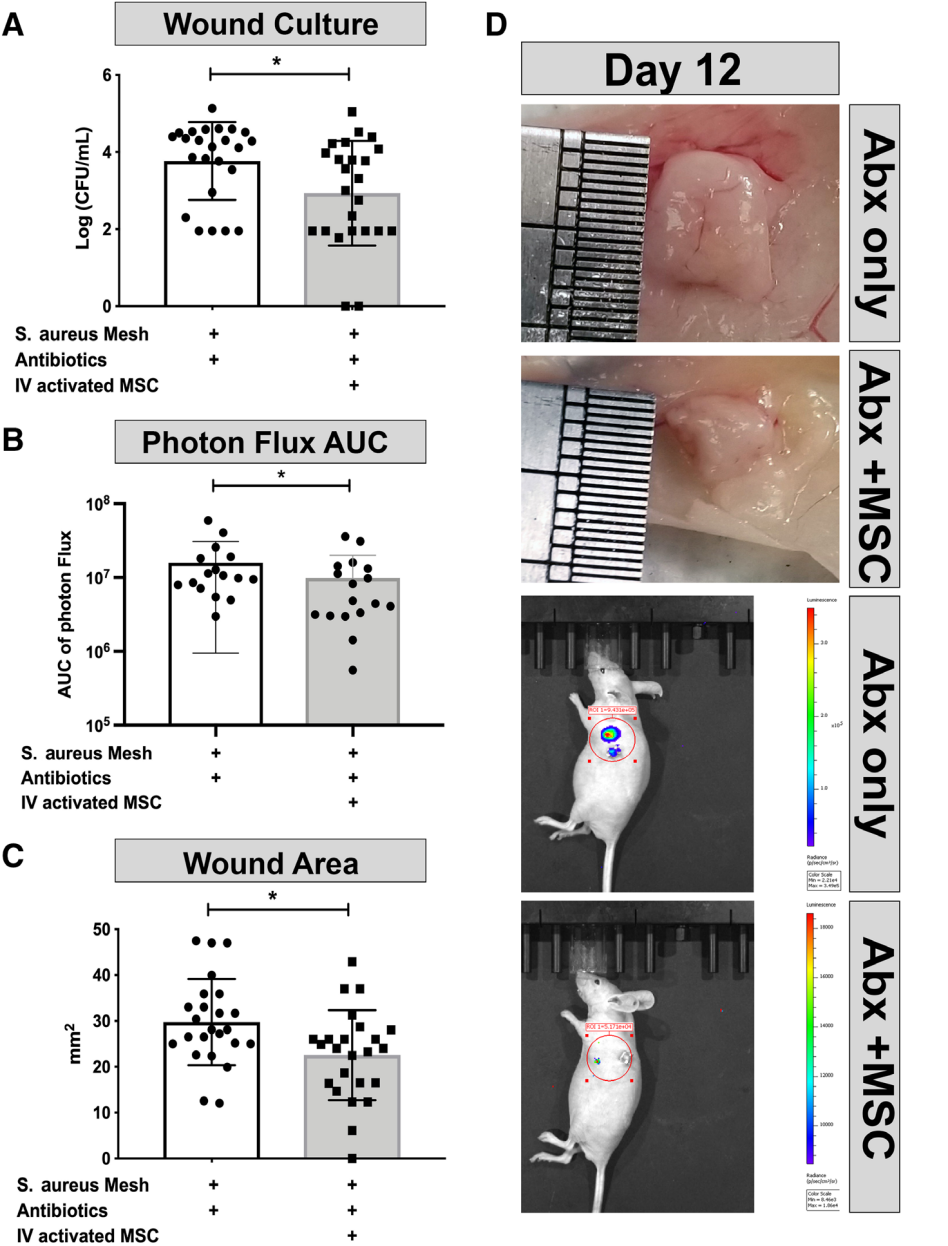
Treatment of chronic biofilm infection by activated mesenchymal stem cells (MSC). Mice (n = 6 per group) were implanted with *S. aureus* infected mesh, then treated with activated MSC and amoxi‐clav, or amoxi‐clav only, as described in the Materials and Methods section. A, Bacterial bioburden in wound tissues (CFU/wound tissue) at the end of the 12 day study. Results pooled from four independent experiments. B, Luminescent imaging of wound bioburden, determined using an IVIS unit, and converted to area under the curve (AUC) Results pooled from three independent experiments. C, Mean measured wound area (mm^2^) for each treatment group of mice. Results pooled from four independent experiments. D, Representative digital camera images of wound tissues immediately following euthanasia, obtained from treated and control mice. Lower two images showing representative IVIS imaging from treated and control mice. With red circle showing the field used to calculate radiance in ROI, radiance color scale shown in right bar. * denotes *P* < .05 as assessed by two tailed nonparametric *t* test and Mann‐Whitney post‐test

We previously reported that activated mouse MSC were able to effectively control and eliminate biofilm infection in a mouse model when coadministered with a beta‐lactam antibiotic (amoxi‐clav).^4^ However, it has not been determined previously whether human MSC exhibit similar activity against chronic *S. aureus* biofilm infections. To address this question, *nu/nu* mice were implanted with *S. aureus* coated surgical mesh as previously described^4^ and then treated with a series of four i.v. injections of pIC activated MSC at 3‐day intervals, together with oral daily administration of amoxi‐clav. The dose of human MSC used for this study was chosen based results from previous study^4^ which showed efficacy in bacterial clearance using mouse MSC in the same model of chronic biofilm infection. The bacterial burden on and around the implanted mesh material was determined on day 12 by direct culture of sonicated wound tissues. We observed that bacterial CFUs were significantly reduced in mice treated with activated MSC, compared with animals treated with antibiotics alone (Figure 7A). Quantitation of bacterial burden using luciferase imaging gives similar results (Figure 7B). In addition, the overall wound surface area was significantly smaller in the MSC‐treated group compared with control animals (Figure 7C), decreasing from an average diameter of 29.8 mm^2^ to 22.5 mm^2^. There was also less purulent material within the abscesses of MSC treated animals, rendering the mesh material more readily visualized (Figure 7D). These results indicate therefore that activated human MSC are also effective in reducing chronic bacterial infections, similar to what has been reported previously for activated canine and murine MSC.^4^

## 4 DISCUSSION

MSC have been widely evaluated experimentally and clinically for their ability to stimulate wound healing and suppress inflammation.^36^, ^37^, ^38^ However, there is comparatively less research regarding the use of MSC for anti‐infective therapy.^4^, ^21^ In particular, even less is known regarding the use of MSC for treatment of chronic bacterial infections, which typically involve the formation of bacterial biofilms and high levels of antimicrobial resistance.^4^, ^5^, ^39^

Therefore, in the present study, which follows a prior publication by our group demonstrating the utility of activated MSC for treating chronic MDR bacterial infections^4^; we have now examined in detail the direct and indirect antimicrobial properties of human MSC. Notably, these studies confirmed and extended our understanding of the direct antibacterial activity of MSC reported previously, and also elucidated new indirect mechanisms involving activation of host innate immune defenses. These host defenses included MSC‐induced augmented NET formation by neutrophils. Although much of the direct antibacterial activity of MSC involving production of AMPs has been previously reported, little has been noted previously regarding the ability of MSC to disrupt preexisting bacterial biofilms.^39^ Here, we also report that newly forming or already formed biofilms can be disrupted by MSC, highlighting the potential for MSC to be used as a treatment for chronic infections, including those often associated with biofilms.^40^, ^41^ We also found that MSC produced factors that enhance the bactericidal activity of all major classes of antibiotics evaluated, including enhancement of antibiotic activity against MDR bacteria.

Production of AMP is one of the most widely investigated direct antimicrobial mechanisms of MSC.^5^, ^18^, ^20^, ^21^, ^42^ Previous studies have reported spontaneous production of several different AMP by human and animal MSC, as revealed by bactericidal assays.^18^, ^21^ Although most studies have reported that MSC constitutively produce antimicrobial factors, there are conflicting reports as to whether MSC activation with TLR ligands or cytokines enhances AMP production and bactericidal activity.^5^, ^42^ In our studies, which investigated the effects of MSC activation on transcription of AMP genes and induction of bactericidal activity, we observed no net increase in in vitro bactericidal activity over that generated by nonactivated MSC. Thus, we concluded that the overall net effect of MSC activation with innate immune stimuli was not reflected by increased bactericidal activity; and production of antimicrobial factors appears to be a constitutive property of MSC.

We also discovered several important indirect mechanisms by which MSC may generate antibacterial activity in vivo, including induction of NET formation by neutrophils and increased neutrophil phagocytosis. It is also important to note that activation of MSC with TLR ligands, particularly the TLR3 ligand pIC, enhanced production of factors secreted by MSC which augmented host innate immune responses to bacterial infections. For example, exposure of neutrophils to activated MSC CM elicited significantly greater bacterial phagocytosis than CM from nonactivated MSC (Figure 5). These results are consistent with those of Brandau et al, who also reported that neutrophil phagocytosis was enhanced following exposure to CM from LPS‐activated MSC.^43^ The same group also reported increased survival of neutrophils following exposure to MSC CM.^44^

We also discovered that CM from activated MSC triggered a significant increase in neutrophil NET area formation, compared with CM from nonactivated MSC or to control neutrophils exposed only to *S. aureus*. This is an important effect of MSC, because formation of NETs is an important mechanism by which neutrophils can contain and eliminate bacterial infections.^11^ Thus, activation of MSC appears to be an essential step in maximizing the interaction of MSC with host innate immune defenses to increase bacterial killing. One possible mediator of increased NET formation in response to MSC CM is IL‐8^45^, which has been reported as a key inducer of NET.^46^ Thus, our finding that pIC activation triggered increased secretion of IL‐8 by MSC is consistent with a possible IL‐8 mechanism for NET formation.

In summary, we have shown here that human MSC are capable of killing drug‐resistant bacteria in the setting of chronic infection, as well as the disruption of established biofilms and prevention of biofilm formation. The effectiveness of TLR activated MSC as a new therapeutic for coadministration with conventional antibiotics was demonstrated in vivo in a mouse chronic *S. aureus* biofilm model. These findings suggest that MSC could be administered therapeutically to patients being concurrently treated with most antibiotics with the expectation of enhanced antibiotic activity, and that there would not be limitations on concurrent MSC treatment based on a particular class of antibiotic. Notably, we also described new mechanisms by which activated MSC interacted with host innate immune defenses, which provides additional understanding of how activated MSC can be used therapeutically for treatment of chronic bacterial infections. Thus, there is reason to consider the use of activated MSC as a novel alternative treatment to augment conventional antibiotic therapy for treatment of chronic MDR infections of implants, soft tissues, and bone tissues.

## CONFLICT OF INTEREST

S.D. declared patent ownership on a patent covering antimicrobial stem cell technology. All of the other authors declared no potential conflict of interest.

## AUTHOR CONTRIBUTIONS

L.C.: conception and design, collection and assembly of data, data analysis and interpretation, manuscript writing; V.J.: conception and design, provision of study material or patients, collection and assembly of data; R.I.: administrative support, collection and assembly of data, data analysis and interpretation; J.C.: assembly of data, data analysis and interpretation; A.S.: collection and/or assembly of data, data interpretation; S.D.: conception and design, provision of study material or patients, data analysis and interpretation, manuscript writing, final approval of manuscript.

## Data Availability

The data that support the findings of this study are available from the corresponding author upon reasonable request.
